# Improving imbalance classification via ensemble learning based on two-stage learning

**DOI:** 10.3389/fncom.2023.1296897

**Published:** 2024-01-05

**Authors:** Na Liu, Jiaqi Wang, Yongtong Zhu, Lihong Wan, Qingdu Li

**Affiliations:** ^1^Institute of Machine Intelligence, University of Shanghai for Science and Technology, Shanghai, China; ^2^Origin Dynamics Intelligent Robot Co., Ltd., Zhengzhou, China

**Keywords:** imbalance, prior shift, covariate shift, reweighting, logit adjustment

## Abstract

The excellent performance of deep neural networks on image classification tasks depends on a large-scale high-quality dataset. However, the datasets collected from the real world are typically biased in their distribution, which will lead to a sharp decline in model performance, mainly because an imbalanced distribution results in the prior shift and covariate shift. Recent studies have typically used a two-stage learning method consisting of two rebalancing strategies to solve these problems, but the combination of partial rebalancing strategies will damage the representational ability of the networks. In addition, the two-stage learning method is of little help in addressing the problem of covariate shift. To solve the above two issues, we first propose a sample logit-aware reweighting method called (SLA), which can not only repair the weights of majority class hard samples and minority class samples but will also integrate with logit adjustment to form a stable two-stage learning strategy. Second, to solve the covariate shift problem, inspired by ensemble learning, we propose a multi-domain expert specialization model, which can achieve a more comprehensive decision by averaging expert classification results from multiple different domains. Finally, we combine SLA and logit adjustment into a two-stage learning method and apply our model to the CIFAR-LT and ImageNet-LT datasets. Compared with the most advanced methods, our experimental results show excellent performance.

## 1 Introduction

Benefiting from the development of computing resources in recent years, deep neural networks (DNNs) have been widely used in image classification (He et al., 2016), image segmentation (Zhou et al., 2019), object detection (Tian et al., 2019), etc. These successful application cases usually require large-scale high-quality labeled data, such as ImageNet (Russakovsky et al., 2015) and COCO (Lin et al., 2014), in which the sample distribution in the training and test dataset is almost consistent. However, training datasets collected from the real world generally have a biased distribution, i.e., the number of samples of each class varies greatly. Models trained by biased datasets will not only cause minority class samples to be misidentified as majority class samples but also confuse minority class samples with hard samples from the majority class, eventually leading to a sharp drop in network performance.

The prior shift and covariate shift resulting from an imbalanced distribution are the primary causes of the decline in network performance. Prior shift refers to the phenomenon that the label distribution of one class in the training dataset and test dataset is inconsistent. Covariate shift mainly refers to the phenomenon that the data distribution of one class in the training dataset and test dataset is inconsistent. These shifts make the network parameters overfit to some majority class samples, resulting in the model's overconfidence in these examples and poor performance on the test dataset. For a long time, many studies have concentrated on developing rebalancing strategies to alleviate this overfitting, such as reweighting for the loss function (Ren et al., 2018; Cui et al., 2019), resampling for the training sample (Pouyanfar et al., 2018; Zhou et al., 2020), and logit adjustment for output logit (Menon et al., 2021; Xu et al., 2021). These strategies provide some good ideas for solving the problems caused by the imbalanced distribution. However, although reweighting and resampling can address class imbalance issues to some extent, the direct application of these methods will damage the deep feature representation ability of the network, making it difficult for the network parameters to reach their theoretical optimal solution (Zhou et al., 2020).

Adopting a two-stage learning strategy, typically using two separate rebalancing strategies in two training stages to decouple network feature representation learning and classifier learning, is a common way to overcome the issues mentioned above. However, some rebalancing strategies are incompatible, e.g., using resampling in the first stage and reweighting in the second stage. Reweighting promotes classifier learning, which encourages the classifier's decision boundary to move in the direction of classifying the minority classes as correctly as possible. Resampling ensures that the label distribution of the mini-batches sampled from the training dataset is consistent with the label distribution of the test dataset. Owing to the undersampling of the majority class samples and the oversampling of the minority class samples, some samples are not involved in the training process, resulting in a negative impact on feature representation learning. It is difficult to use the reweighting method to optimize the classifier when the separability of the feature is weak (Zhou et al., 2020). Based on the above analysis, we propose to use data augmentation instead of resampling in the first stage to maximize the representation ability of the network.

**Figure 1:**
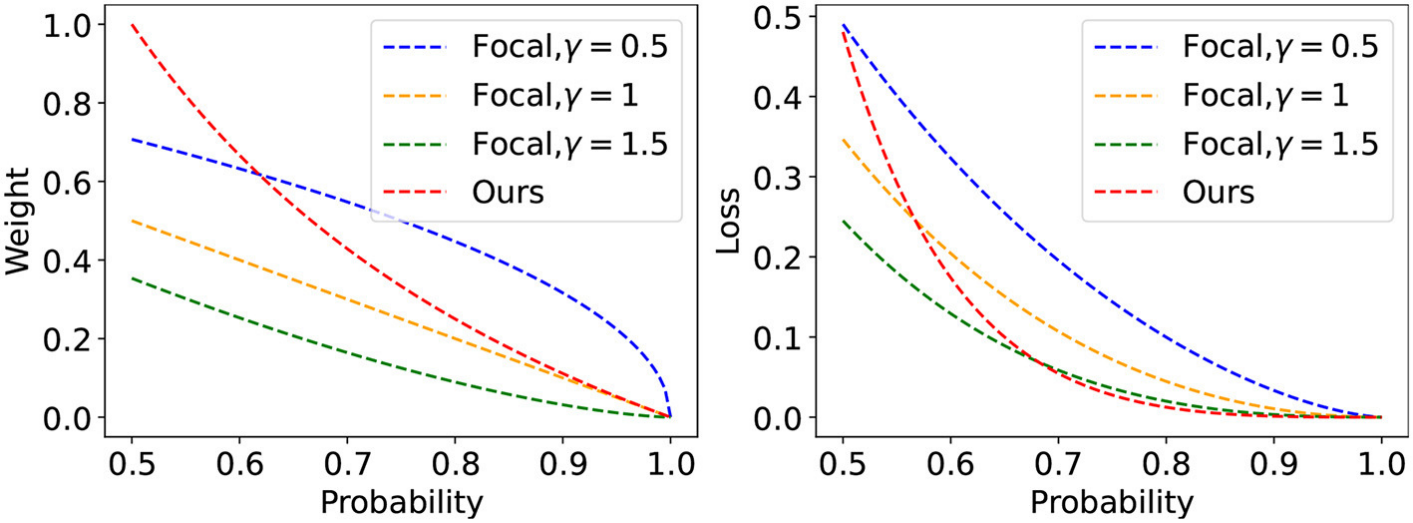
In the process of reweighting based on probability, some hard samples from the majority class will have similar weights to the samples from the minority class. As shown in the curve on the right of the above figure, our method can effectively focus on the hard sample of each class, and the loss of our proposed method rapidly decays in the low probability areas.

Our goal in this work is to design an efficient and useful two-stage learning method using currently available rebalancing strategies. Owing to the conflict between reweighting and resampling, we investigate the effects of the combination of logit adjustment and reweighting on DNNs. We discover that the network performance will be degraded when combining the existing classic reweighting methods with logit adjustment. This is because both logit adjustment and reweighting try to give minority class samples more attention while giving the majority class samples less attention, ultimately making the performance of the majority class drastically deteriorate. Additionally, because the confidence of majority class hard samples and minority class samples is extremely similar, the sample confidence-based reweighting method [such as focal loss (Lin et al., 2017)] will unfairly assign weights to these samples, which will increase the expected calibration error of the network (Guo et al., 2017). To this end, we propose a logit-aware reweighting method (called SLA) that could use the sample with the largest logit of each class as the benchmark sample to assign appropriate weights to the remaining samples (Figure 1).

Furthermore, two-stage learning methods are ineffective at dealing with the covariate shift problem, which is an unavoidable but easily neglected issue in imbalanced image classification. It is hard to ensure that the distribution of the training and test dataset is entirely consistent. The minority class may have dramatically different numbers on the training and test datasets when the distribution of the training dataset is imbalanced, which exacerbates the inconsistency between the training data distribution and testing data distribution. In this situation, it is difficult to train a model with good generalizability using just a two-stage learning method. Inspired by ensemble learning, we propose a multi-domain expert specialization model to enhance the feature extract ability in a specific data distribution. In particular, in the first training stage, three different levels of data augmentation were employed to specialize the original data distribution into three distinct data distributions. Additionally, mixup was used to blend the original smaller feature distribution space into a larger feature space, thereby enhancing the model's feature extraction ability. At the same time, the model also includes a two-stage training loss strategy, which can promote the classifier to learn a more reliable decision boundary. Under the guidance of the two-stage learning method, our proposed model demonstrated excellent performance on existing imbalanced datasets.

In summary, our main contributions are as follows:

(1) For two-stage learning methods, we indicate that the combination of existing reweighting methods and logit adjustment will lead to performance degradation for the majority class or cause significant calibration errors.
(2) We propose a new reweighting method that can repair the weight of the majority of hard samples and minority samples calculated by the sample confidence-based reweighting method without significantly reducing the majority accuracy.
(3) We propose a new ensemble learning framework that provides three deep specialized feature extractors for three different levels of data augmentation, which can significantly improve the representation ability of the network. Under the guidance of our proposed two-stage training loss strategy, it can significantly increase classification accuracy and reduce expected calibration error.

## 2 Related Work

### 2.1 Reweighting

The reweighting method assigns weights to each class or sample to alleviate the model performance degradation caused by imbalanced data. A weighting function that maps the loss function (or gradient) to each sample can be used to determine the weight. Through artificial prior knowledge or a simple neural network, the weighting function could be easily estimated.

Initially, Huang et al. (2016, 2019) used the reciprocal of class frequency as a weighting factor applied to class loss (Wang et al., 2017). Subsequently, Lin et al. (2017) extended the class frequency from a fixed prior to an adjustable parameter version. Khan et al. (2019) further extended the weighting method from the class level to the instance level (Cui et al., 2019). Although this approach is effective, the complex parameter adjustment rules are tedious and not universal. In addition, hard samples from the majority class are frequently weighted improperly because they share a lot of similarities with minority class samples in terms of loss values. To solve this problem, Ren et al. (2018) and Shu et al. (2019) proposed a robust weighted function mapping from samples to instance losses based on the meta-learner. However, it is difficult to estimate the parameters of the weighting network in the meta-learning method. The meta-learning method requires nested training, which costs a lot of time. Also, meta learners need a meta dataset that is close to the distribution of test dataset (Finn et al., 2017; Shu et al., 2019; Jamal et al., 2020; Li et al., 2021). Zhang and Pfister (2021) adjusted the process of meta-learning, which greatly reduced the training cost of meta-learning and alleviated the excessive dependence on metadata distribution. Although meta-learning is currently the best reweighting method for specific datasets, its demanding prerequisites and high training cost precluded us from using it to search for a weighting function.

### 2.2 Logit adjustment

The idea of logit adjustment was expressed earlier as margin loss. The essence of margin loss is to apply margin to logits of a specific class to obtain a greater classification interval (Liu et al., 2016, 2017; Wang et al., 2018). To address the imbalance image classification task, LDAM (Cao et al., 2019), EQL (Tan et al., 2020), and BALMS (Ren et al., 2020) suggest that minority classes need a large margin while majority classes need a small margin, and the margin is determined by an optimal trade-off boundary (Cao et al., 2019) or by using a meta learner (Jamal et al., 2020; Ren et al., 2020). Menon et al. (2021) summarizes the previous margin-based method and proposes the concept of logit adjustment. To find a suitable logit adjustment method more effectively and quickly, adding label distribution as prior information to the logit has become a stable improvement method (Hong et al., 2021; Menon et al., 2021; Xu et al., 2021; Aimar et al., 2022).

### 2.3 Two-stage learning

The two-stage training method usually defers the use of the rebalancing strategy, such as reweighting or resampling, to the second stage (Hong et al., 2021). By using a smaller learning rate, the classifier of the model can obtain a better decision boundary on the feature extracted by the feature extractor. Although the two-stage learning method can achieve decoupling training and improve the generalization performance of the model, combining two conflicting rebalancing strategies will lead to a decrease in model performance (Zhou et al., 2020). Therefore, it is important to carefully select and evaluate different rebalancing strategies to ensure that they are compatible with each other and can lead to improved overall performance. In this study, we found that the combination of logit adjustment and the existing reweighting method causes conflicts, making it difficult for the model to converge to the optimal solution. Based on the above findings, we propose a new reweighting method to address this issue.

## 3 Analysis

For a multi-class classification task, we assume a dataset with *N* samples, in which *X* = {*x*_1_, *x*_2_, ..., *x*_*N*_} denotes the samples and *Y* = {*y*_1_, *y*_2_, ..., *y*_*N*_} denotes the labels. The dataset can be defined as *D* = {(*x*_*i*_, *y*_*i*_), 1 ≤ *i* ≤ *N*}, where *x*_*i*_ denotes the *i*-th sample and $y_{i}\in {\left\{0,1\right\}}^{c}$ is a *c* dimension vector. Our goal is to train a network that can minimize the misclassification error, i.e., $\min\overset{N}{\underset{i=1}{\sum }}\mathbb{P}\left(y_{i}\neq \arg\max p_{y_{i}}\left(x_{i}\right)\right)$, where *p*_*y*_*i*__(*x*_*i*_) represents the probability of *x*_*i*_ belonging to class *y*_*i*_. In general, we use the *softmax cross-entropy* (CE) to represent this error,


(1) $$\begin{array}{l} \ell (y_{i},f(x_{i}))=-\log\frac{\exp(f_{y_{i}}(x_{i}))}{\sum_{j=1}^{c}\exp(f_{y_{j}}(x_{i}))} \end{array}$$

where *f*_*y*_*i*__(*x*_*i*_) and *f*_*y*_*j*__(*x*_*i*_) represent the output logit of *x*_*j*_ belonging to classes *y*_*i*_ and *y*_*j*_. For the class imbalance problem, the direct use of the CE loss function may lead to the bias toward majority classes during the training process and neglect the learning of minority classes, resulting in some minority class samples being mistakenly classified as the majority classes during the testing phase. To address this issue, most reweighting methods usually apply a learnable or pre-designed weighting factor *w* to modulate the CE loss function, which can improve the contribution of minority classes to the average loss and make network learning more focused on minority classes. The reweighting loss function can be expressed by the following equation,


(2) $$\begin{array}{l} \ell (y_{i},f(x_{i}))=-w_{i}\log\frac{\exp(f_{y_{i}}(x_{i}))}{\sum_{j=1}^{c}\exp(f_{y_{j}}(x_{i}))} \end{array}$$

However, it is challenging to derive an explicit reweighting function without prior knowledge. In most reweighting methods, the weighting factor is naturally defined as a small weight for the majority class and a large weight for the minority class. Although this logical viewpoint is empirically correct, it does not consider the imbalanced distribution within the class; the samples of the same class can also be divided into the common sample and rare sample.

### 3.1 Compensation training classifier

From the perspective of data distribution, we can rapidly identify why the model trained from the training dataset often performs poorly in the test phase in imbalance image classification tasks. The training and test objectives can be expressed by the following probability,


(3) $$\begin{array}{l} {\mathbb{P}}^{s}\left(y|x\right)\propto \frac{{\mathbb{P}}^{s}\left(x,y\right)}{{\mathbb{P}}^{s}\left(x\right)}\propto {\mathbb{P}}^{s}\left(x|y\right){\mathbb{P}}^{s}\left(y\right) \end{array}$$

(4) $$\begin{array}{l} {\mathbb{P}}^{t}\left(y|x\right)\propto \frac{{\mathbb{P}}^{t}\left(x,y\right)}{{\mathbb{P}}^{t}\left(x\right)}\propto {\mathbb{P}}^{t}\left(x|y\right){\mathbb{P}}^{t}\left(y\right) \end{array}$$

where *s* represents the source domain (training dataset) and *t* represents the target domain (test dataset). According to Equations (3) and (4), we can further express it as a form of measuring the difference between the training and testing object (Jamal et al., 2020),


(5) $$\begin{array}{l} {\mathbb{P}}^{s}\left(y|x\right)={\mathbb{P}}^{t}\left(y|x\right)\frac{{\mathbb{P}}^{s}\left(x|y\right){\mathbb{P}}^{s}\left(y\right)}{{\mathbb{P}}^{t}\left(x|y\right){\mathbb{P}}^{t}\left(y\right)} \end{array}$$

(6) $$\begin{array}{l} \underset{Covariate\;shift}{\underset{︸}{{\mathbb{P}}^{s}\left(x|y\right)⊖{\mathbb{P}}^{t}\left(x|y\right)}}\;\text{and}\;\underset{Prior\;shift}{\underset{︸}{{\mathbb{P}}^{t}\left(y\right)⊖{\mathbb{P}}^{s}\left(y\right)}} \end{array}$$

Covariate shift is a common issue in deep learning tasks that refers to the situation in which the input data or feature distribution differs between the training dataset and test dataset, leading to a poor generalization performance of the trained model on the test dataset. The network will inevitably suffer from this damage during training. For the imbalance image classification task, this damage will become more serious (Jamal et al., 2020). Prior shift refers to a common problem that arises when there is some difference in the label distribution between the training and test datasets. Specifically, it is caused by the difference in the distribution of the number of samples per class between the training and test datasets (Menon et al., 2021). This makes the algorithm learn a biased representation, resulting in decreased performance when applied in the test phase. Owing to the difficulty in estimating covariate shift, we will discuss strategies for mitigating this problem in Section 4.2, but temporarily ignore its impact here. In previous training processes, the softmax classifier was typically used for both training and testing. However, as indicated by Equation (6), two shifts between the training and test objectives exist. To address these problems, we can adjust the training loss as follows:


(7) $$\begin{array}{l} \ell (y_{i},f(x_{i}))=-\log\frac{\exp(f_{y_{i}}(x_{i})+\log\mu_{i})}{\sum_{j=1}^{c}\exp(f_{y_{j}}(x_{i})+\log\mu_{j})} \end{array}$$

where $\mu_{i}=\frac{{\mathbb{P}}^{train}\left(y_{i}\right)}{{\mathbb{P}}^{test}\left(y_{i}\right)}$, μ is a factor to measure the label distribution difference between the training and test datasets. Furthermore, Equation (7) can be expressed as follows:


(8) $$\begin{array}{l} \ell \left(y_{i},f\left(x_{i}\right)\right)=-\log\left[1+\underset{j\neq i}{\sum }\frac{\mu_{j}}{\mu_{i}}\exp\left(f_{y_{j}}\left(x_{i}\right)-f_{y_{i}}\left(x_{i}\right)\right)\right] \end{array}$$

If *y*_*i*_ represents the majority classes and μ_*j*_ < μ_*i*_, the loss value calculated based on Equation (8) will decrease compared with CE. This will make the network tend to learn from minority classes during parameter updates, reducing the attention to majority classes, thereby improving the performance of the network. For convenience, we will use logit adjustment (LA) to represent the above training losses.

### 3.2 Mixed reweighting and LA

Compensating the output logit can effectively alleviate the learning bias caused by imbalanced data distribution. To further improve the effectiveness of boundary correction, we combine reweighting with LA into a new paradigm and explore effective combination strategies. Specifically, we conduct experiments using ResNet-32 trained on the CIFAR-100-LT dataset with different combinations of reweighting and LA. The reweighting methods, which include reweight (RW) (Wang et al., 2017), class-balanced loss (CB) (Cui et al., 2019), and focal loss (FL) (Lin et al., 2017), were introduced in the 180th epoch (out of a total of 200 epochs) for ResNet-32.

Table 1 presents the results obtained from the aforementioned settings. We can infer that (1) the combination of existing reweighting methods and LA will lead to a decline in overall accuracy, especially in the majority classes. This indicates that there is a conflict between the existing reweighting and LA, and there is an overlap between providing large margins and large weights for the minority classes, which ultimately leads to a significant decline in the performance of the majority classes. (2) Although focal loss can maintain the accuracy of the majority classes to a certain extent, it is expected that the calibration error is still large. This is because focal loss assigns similar weights to the hard samples from majority classes and the samples from minority classes.

**Table 1:** Top-1 accuracy (%) and ECE (%) from the different combinations of logit adjustment and different reweighting methods.

**Method**	**Many**	**Medium**	**Few**	**All**
LA	64.9	50.3	29.5	49.5/3.8
LA + RW	30.8	39.5	23.7	31.8/36.9
LA + CB	44.8	44.1	42.0	43.8/4.4
LA + FL	62.5	49.9	32.8	49.5/3.2

We used the mixup α = 0.4 on the CIFAR-100-LT dataset (ρ = 100).

## 4 Method

### 4.1 Sample logit-aware reweighting

The purpose of the two-stage training method is to focus on obtaining a powerful feature extractor and classifier in the first stage and reduce the difference between the sample confidence and the overall class confidence in the second stage. From the perspective of sample confidence, assigning higher weights to samples with low confidence is an effective solution. However, when it comes to hard samples in the majority classes, their confidence levels are often indistinguishable from the samples in the minority classes. To overcome this issue, we propose a sample logit-aware reweighting method (called SLA in this study) that reduces the gap between the single sample confidence and the overall class average confidence, without significantly sacrificing accuracy. The sample confidence can be calculated as follows:


(9) $$\begin{array}{l} p_{i}=\frac{\exp(f_{y_{i}}(x_{i})+\log\mu_{i})}{\sum_{j=1}^{c}\exp(f_{y_{j}}(x_{i})+\log\mu_{j})} \end{array}$$

where *p*_*i*_ represents the predicted probability that sample *x*_*i*_ belongs to the correct label after adjusting for the output logit. In addition, based on the idea of SLA, to make the weighting factor *w*_*i*_ pay more attention to hard samples based on the probability reweighting method, we use the sample with the maximum logit of each class to guide the learning of the remaining samples. The sample weight can be expressed as follows:


(10) $$\begin{array}{l} w_{i}={\left(1-p_{i}\right)}^{\gamma }\exp\left(f_{y_{i}}\left(x_{*}\right)-f_{y_{i}}\left(x_{i}\right)\right) \end{array}$$

where x_*_ is the sample with the largest logit in all training samples belonging to *y*_*i*_, and γ is a weighted rate adjustment factor. Commonly, *f*_*y*_*i*__(*x*_*i*_) = *W*_*y*_*i*__*z*_*i*_, *W* is the weight matrix of the linear layer and *z*_*i*_ is the feature embedding of *x*_*i*_. To obtain more stable sample weights, we calculate the cosine value by standardizing *W*_*y*_*i*__ and *z*_*y*_*i*__.


(11) $$\begin{array}{l} \cos_{\theta }\left(y_{i}\right)=\frac{W_{y_{i}}^{T}}{∥W_{y_{i}}∥}\cdot \frac{z_{i}}{∥z_{i}∥} \end{array}$$

**Figure 2:**
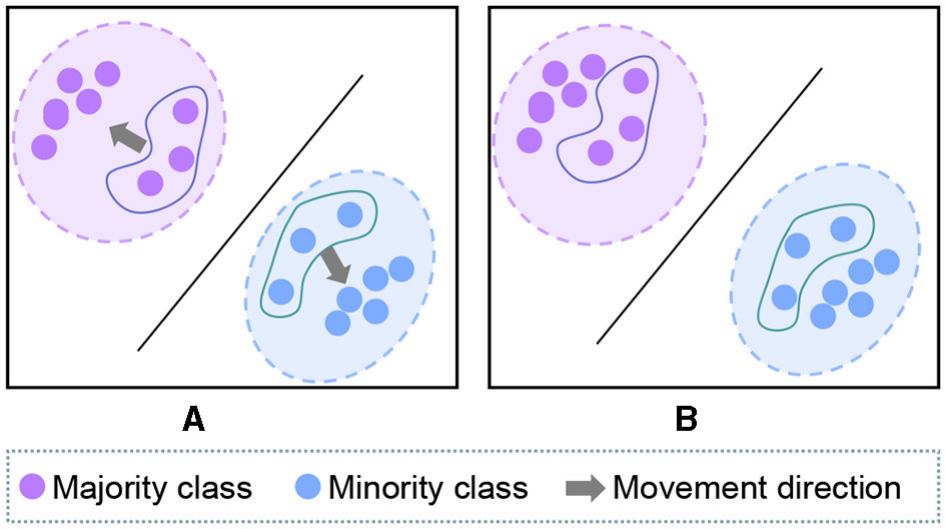
**(A)** The hard samples from the majority class and some samples from the minority class are very close between the decision boundary. **(B)** SLA can allow these samples to converge toward their class center, thereby improving accuracy and reducing ECE.

Therefore, after transforming the logit into the corresponding cosine representation (Figure 2), the final SLA reweighting formula can be expressed as follows:


(12) $$\begin{array}{l} w_{i}={\left(1-p_{i}\right)}^{\gamma }\exp\left(\tau \cos\theta_{y_{*}}-\tau \cos\theta_{y_{i}}\right) \end{array}$$

where θ_*y*_*i*__ corresponds to the angle between *z*_*i*_ and *W*_*y*_*i*__, θ_y_*__ corresponds to the z_*_ and *W*_*y*_*i*__, and τ is a hyperparameter.

### 4.2 Multi-domain expert specialization model

The main objective of the first stage of training in the two-stage method is to enhance the feature extraction capability of the network. However, it is challenging for a single-channel feature extractor to learn robust parameters when the data distribution is extremely imbalanced, particularly when complex data augmentation techniques are applied. To address this problem, we propose a multi-domain expert specialization model for augmenting data across multiple domains (Algorithm 1).

**Algorithm 1:** The training process of our proposed method.

Input: Training data $D={\left\{\left(x_{i},y_{i}\right)\right\}}_{i=1}^{N}$, batch size *n*.
Output: Optimized network parameters θ.
1: Initialization for θ
2: while t < = MaxEpoch **do**
3: ${\left\{\left(x_{i},y_{i}\right)\right\}}_{i=1}^{n}$ ← Sample a minibatch from *D*.
4: Getting $\left\{\left(\tilde{x_{i}},\tilde{y_{i}}\right)\right\}$ using Equation (14)
5: Getting each expert loss using Equation (16)
6: Calculate total loss using Equation (18)
7: if t = = SwitchEpoch **then**
8: Create a list *S* to store cosθ_y_*__ of each class.
9: end **if**
10: if t > SwitchEpoch **then**
11: Find the sample with the largest logit score for each class and calculate cosθ_y_*__ using Equation (11)
12: Update the list *S*_*t*_→*S*_*t*+1_.
13: end **if**
14: Use SGD to update network parameters θ;
15: end **while**

#### 4.2.1 Multiple data augment header with mixup

**Figure 3:**
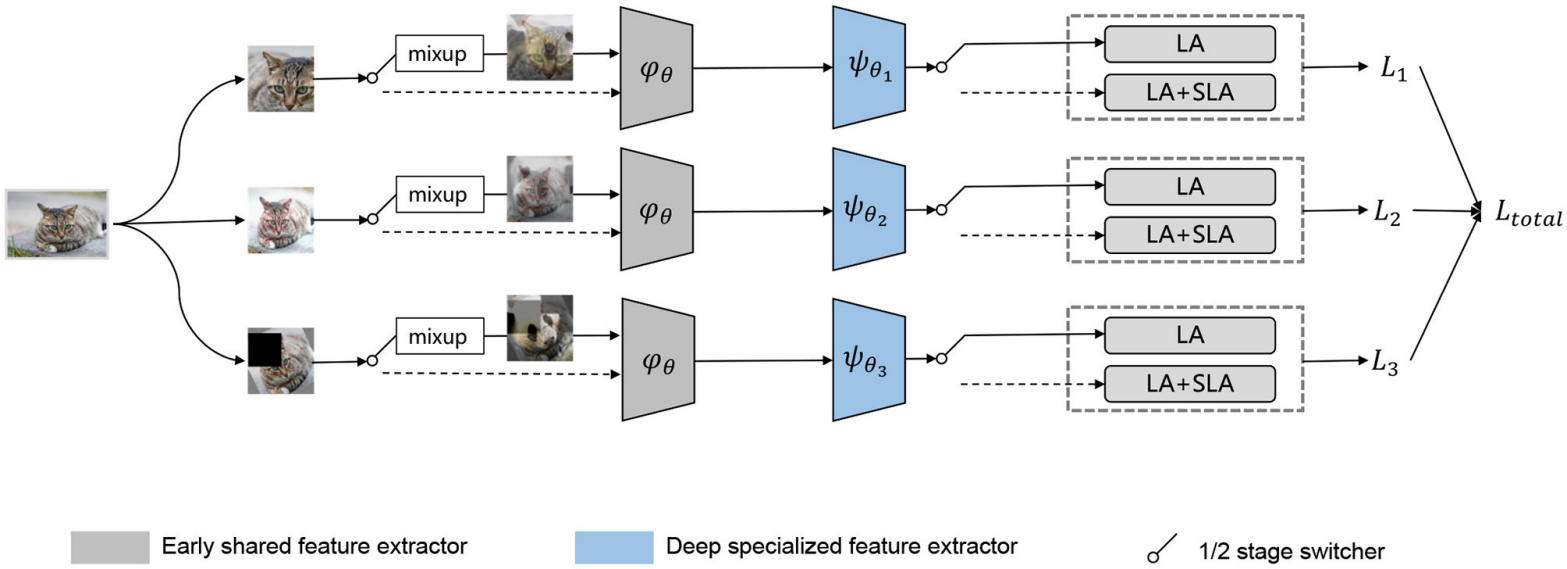
An overview of our proposed two-stage learning method is as follows: in the first stage, we employ LA to train a robust feature extractor by learning feature representations of each class on a larger feature space that is guided by the mixup technique. In the second stage, we introduce the SLA reweighting method and remove augmentation and mixup to optimize the decision boundary of the classifier.

Before inputting data into the network, multiple data augmentation techniques (including mixup) should be applied to the data. The purpose of data augmentation is to expand the domain boundary of the source domain data as much as possible (Figure 3), thereby alleviating the severe covariate shift caused by the imbalanced distribution.


(13) $$T_{k}(x_{i})=\left\{ \begin{array}{l} \text{mixup}({\text{Aug}}_{k}(x_{i}))\text{ if 1-stage}\\ {\text{Aug}}_{k}(x_{i})\text{ if 2-stage} \end{array}\right.$$

where *T*_*k*_ represents the result of applying the *k*-th data augmentation function (Aug) to input *x*_*i*_. To make better use of data augmentation, we apply the mixup strategy based on the augmented data during the first stage of training. By using mixup, the resulting data can be represented as if it was sampled from a new sampling space: $D_{l}=\left\{\left(\tilde{x_{i}},\tilde{y_{i}}\right)\right\},1\leq i\leq N^{\prime }$. After combining two augmented samples using mixup, the newly generated sample $\left\{\tilde{x},\tilde{y}\right\}$ can be expressed as follows:


(14) $$\begin{array}{l} \tilde{x}=\epsilon T(x_{i})+(1-\epsilon )T(x_{j})\\ \tilde{y}=\epsilon y_{i}+(1-\epsilon )y_{j} \end{array}$$

where ϵ~*Beta*(α, α) with α∈(0, 1), which allows for flexible adjustment of the mixing ratio during training. By introducing this sampling procedure, the model can be trained on a new sample space that comprises mixtures of the original augmented inputs, allowing it to learn more robust representations and improve its ability to generalize to new samples.

#### 4.2.2 Early shared and deep special feature extractor

During the feature extraction process in the early layers of CNN, the network tends to learn low-level features such as points and lines. As a result, we opt for utilizing the same early shared feature extractor for different enhanced data during the first stage. However, during deep feature extraction, the varying enhancement of three levels of data augmentation requires specialized deep feature extractors to extract professional features. To achieve this goal, we employ three distinct deep feature extractors, with their outputs expressed as


(15) $$\begin{array}{l} f^{k}\left(x_{i}\right)=\psi_{\theta_{k}}\left(\varphi_{\theta }\left(T_{k}\left(x_{i}\right)\right)\right) \end{array}$$

where *k*∈[1, 3], $f^{k}\left(x_{i}\right)$ represents the output logit after *x*_*i*_ passes through the early shared feature extractor φ_θ_ and *k*-th deep special feature extractor ψ_θ_*k*__.

#### 4.2.3 Two-stage training loss strategy

As analyzed in Section 3.1, the two-stage training method requires training a better feature extractor in the first stage. Therefore, we only compensate the classifier and do not use any reweighting method during the first stage of training. Hence, the model should use a reweighting method in the following training process to optimize the decision boundary of the classifier to reduce ECE.


(16) $$\begin{array}{l} L_{k}(y_{i},f^{k}(x_{i}))=-w_{i}^{k}\log\frac{\exp(f_{y_{i}}^{k}(x_{i})+\log\mu_{i})}{\sum_{j=1}^{c}\exp(f_{y_{j}}^{k}(x_{i})+\log\mu_{j})} \end{array}$$

Equation (16) represents the loss function *L*_*k*_ for the *k*-th expert, and $w_{i}^{k}$ can be expressed in the following form:


(17) $$w_{i}^{k}=\left\{ \begin{array}{l} 1\text{ if 1-stage}\\ (1-p_{i}^{k}{)}^{\gamma }\exp(\tau \cos\theta_{y_{*}}-\tau \cos\theta_{y_{i}})\text{ if 2-stage} \end{array}\right.$$

Here, γ and τ are hyperparameters, $p_{i}^{k}$ is the predicted probability of the *k*-th expert of the sample *x*_*i*_ belonging to its true class after compensating the out logit, and $f_{y_{i}}^{k}\left(x_{i}\right)$ is the output from the *k*-th expert belongs to *y*_*i*_ class from the *k*-th expert. Thus, the final loss function can be expressed as the weighted sum of losses obtained by three experts. We use ϵ_*k*_ to indicate the degree of attention given to the *k* experts; increasing ϵ_*k*_ can make the model more inclined to learn from expert *k*-th. To make the results of other ensemble learning methods more comparable and ensure the fairness of the comparison, we set ϵ_*k*_ to 1 in all the experiments conducted in this study. The final expression for the total loss function is represented by Equation (18).


(18) $$\begin{array}{l} L_{total}=\epsilon_{1}L_{1}+\epsilon_{2}L_{2}+\epsilon_{3}L_{3} \end{array}$$

#### 4.2.4 Test time prediction

Considering we used a loss function in the training stage that was the weighted sum of individual losses from multiple experts, we employ the weighted average logit output of three experts during the test process as our final prediction to minimize empirical risk. The probability that *x*_*i*_ belongs to a certain class can be calculated using the following formula:


(19) $${\hat{p}}_{i}=\arg\max\{\frac{1}{3}\sum_{k=1}^{3}\epsilon_{k}\frac{\exp(f_{y_{i}}^{k}(x_{i}))}{\sum_{j=1}^{c}\left(f_{y_{j}}^{k}(x_{j})\right)},i\in [1,c]\}$$

## 5 Experiments

### 5.1 Datasets

#### 5.1.1 CIFAR-10-LT and CIFAR-100-LT

The CIFAR-10 and CIFAR-100 datasets are common image classification datasets that contain 50,000 training images and 10,000 test images with 10 or 100 classes (Krizhevsky et al., 2009). Following Cao et al. (2019), we create the long-tailed distribution version by randomly removing training samples and keeping the distribution of the test dataset balanced. We use the imbalance ratio ρ to represent the imbalance degree of the dataset, where ρ = *N*_*max*_/*N*_*min*_, *N*_*max*_(*N*_*min*_) is the number of the most (least) frequent class. In this study, we used the imbalance ratio of 10, 50, 100, and 200 to carry out experiments.

#### 5.1.2 ImageNet-LT

ImageNet (Russakovsky et al., 2015) is a large-scale dataset for object classification. Based on this, Liu et al. (2019) made ImageNet-LT by sampling a subset following the Pareto distribution with power value α = 0.6 from ImageNet, which contains ~115.8K images with 1,000 classes. This choice is crucial because it controls the proportion of frequent and infrequent categories in the long-tailed distribution. In addition, the Pareto distribution has a characteristic long tail, which is desirable as it can generate more extreme long-tail datasets that are closer to real-world scenarios. The number of samples for the most frequent class is 1,280 images, whereas the number of samples for the least frequent class is only five images, i.e., the imbalance ratio ρ = 256.

### 5.2 Evaluation protocol

#### 5.2.1 Expected calibration error

The purpose of model calibration is to ensure that the predictive confidence of the model for one sample is consistent with the true empirical risk probability. Therefore, we use the expected calibration error (ECE) to measure the calibration degree of the network. To compute ECE, we group all *N* predictions into *B* interval bins of equal size. The ECE can be defined as:


(20) $$\begin{array}{l} ECE=\overset{B}{\underset{b=1}{\sum }}\frac{\left|T_{b}\right|}{N}|acc\left(T_{b}\right)-conf\left(T_{b}\right)| \end{array}$$

where *T*_*b*_ is the set of samples with a network prediction belonging to Bin-*b*, *acc*(·) is the accuracy of *T*_*b*_, and *conf*(·) is the predicted confidence of *T*_*b*_.

### 5.3 Implementation details

For CIFAR-10-LT and CIFAR-100-LT datasets, we used ResNet-32 as the benchmark network. We used three different levels of data augmentation; the specific details are shown in Appendix. Following most practices, we set the batch size as 128 and the weight decay as 5e-4. We used the SGD optimizer, and the initial learning rate was 0.1. For all experiments on the main result, the hyperparameter α was set to 0.2, and τ was set to 1. For a fair comparison, we trained 200 and 400 epochs, respectively, based on the above settings. During the training of 200 epochs, the learning rate was decreased by a factor 10 at epochs 160 and 180. During the training of 400 epochs, the learning rate was decreased by a factor 10 at epochs 320 and 360. The 1/2 stage switching time was set to epochs 160 and 320.

For ImageNet-LT, we adopted ResNet-50 and ResNetx-50 as the benchmark networks. As with CIFAR-LT, three different levels of data augmentation were employed. The batch size was set to 128 for ResNet-50 and 64 for ResNetx-50 with the weight decay as 5e-4. We used the SGD optimizer, and the initial learning rate was set at 0.025. We used a cosine annealing learning rate schedule. For all experiments on the main result, the parameter α was set to 0.1, and τ was set to 1. During the training of 180 epochs, the learning rate changed periodically according to the law of the cosine annealing learning rate schedule. The 1/2 stage switching time was set to epoch 160.

### 5.4 Main results

#### 5.4.1 Result for CIFAR-LT

**Figure 4:**
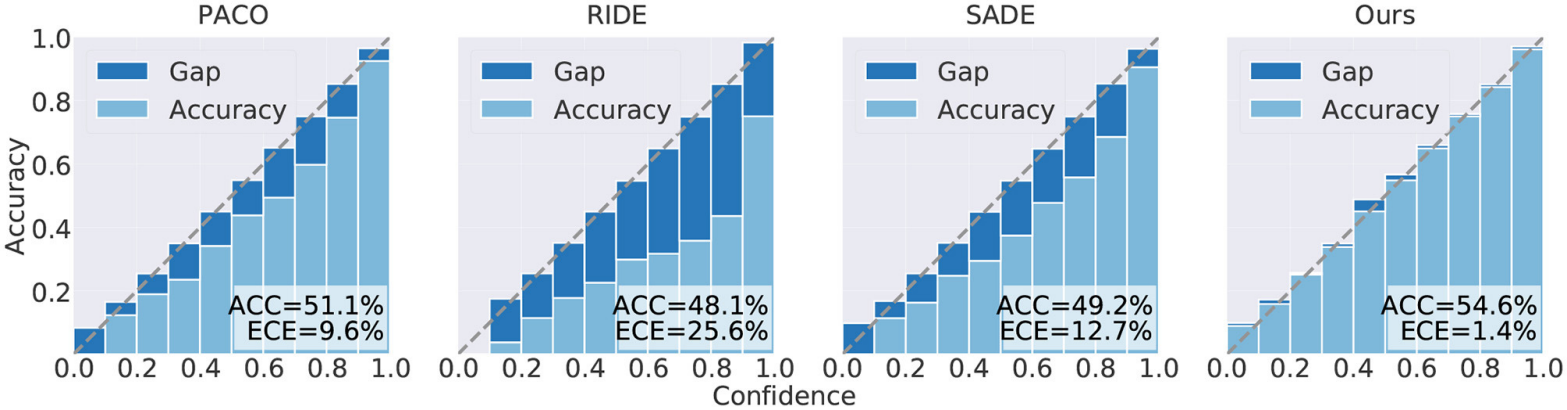
Test accuracy (%) and ECE (%) of different methods trained for 200 epochs on CIFAR-100-LT (ρ = 100), including the contrastive learning method PaCo and the ensemble learning methods RIDE, SADE, and ours.

Table 2 presents a comparison of the results obtained by our proposed method and other various methods on CIFAR-LT. All experiments trained for 200 epochs. First, we observed that our method outperformed existing methods across all class imbalance ratios. Specifically, our proposed method achieved improvements of 4.7, 4.3, 3.2, and 1.4% on CIFAR-10-LT, and 3.9, 4.2, 4.2, and 4.1% on CIFAR-100-LT for imbalance ratios of 200, 100, 50, and 10, respectively, when compared with the state-of-the-art method. Second, it is worth noting that our method maintained a significant performance gap compared with other methods regardless of the class imbalance ratio, which demonstrates the effectiveness of our method. Furthermore, we observed that, compared with existing multi-expert methods, the accuracy gap between our proposed method and theirs gradually decreased with a decrease in the imbalance ratio. This phenomenon can be explained by the fact that when the imbalance ratio is small, data from the minority classes already cover a large data distribution space in the training dataset, thus weakening the effect of data augmentation on alleviating covariate shift caused by an imbalanced distribution. At the same time, we compared the SLA of different methods and the results showed that our proposed method achieved lowest SLA in addition to achieving considerable accuracy (Figure 4).

**Table 2:** Test accuracy (%) on CIFAR-100-LT for various methods with different imbalance ratios ρ.

**Method**	**Dataset**	**CIFAR-10-LT**				**CIFAR-100-LT**			
	**Backbone**	**ResNet-32**				**ResNet-32**			
	**Imbalance ratio**	**200**	**100**	**50**	**10**	**200**	**100**	**50**	**10**
CE		65.7	70.4	74.8	83.4	38.3	38.2	43.9	56.9
CB-Focal (Cao et al., 2019)		–	74.6	79.2	86.8	–	39.6	45.2	58.0
MW-NET (Shu et al., 2019)		–	75.2	80.0	87.8	–	42.1	46.7	58.4
LDAM + DRW (Cao et al., 2019)		–	77.0	81.2	88.2	–	42.0	46.6	58.7
BBN (Zhou et al., 2020)		–	79.8	82.4	88.1	–	42.5	47.2	59.4
LA (Menon et al., 2021)		–	79.9	83.4	89.3	–	43.9	49.8	59.8
Mixup (Zhang et al., 2018)		–	73.3	77.6	87.2	–	39.6	45.1	58.4
Remix + DRW (Chou et al., 2020)		–	79.8	–	89.1	–	46.8	–	61.3
MiSLAS (Zhong et al., 2021)		–	82.1	85.7	90.0	–	47.0	52.3	63.2
RIDE (Zhang et al., 2018)		77.9	81.5	83.4	85.9	44.8	48.5	51.0	57.8
ACE (Cai et al., 2021)		–	81.2	84.3	–	–	49.4	50.7	–
SADE (Zhang et al., 2022)		78.6	82.4	85.6	90.5	46.2	50.4	54.2	63.8
Ours		**83.3**	**86.7**	**88.9**	**91.9**	**50.1**	**54.6**	**58.4**	**67.9**

All experiments used ResNet-32 as the backbone and trained for 200 epochs.

At the same time, we performed long-term training for 400 epochs on CIFAR-100-LT (ρ = 100), and the corresponding results are presented in Table 3. Compared with those in Table 2, our proposed method demonstrated continued improvement in accuracy beyond 200 epochs. This is attributed to the inclusion of multiple data augmentation headers in our network architecture, which significantly enhances the representation ability of the network's feature extractor and mitigates the representation difficulties introduced by covariate shift, leading to enhanced overall accuracy. More importantly, the performance of our proposed method in the few classes is far better than that of other methods. This is because we have assigned a specialized feature extractor for each level of data augmentation, which can prevent the representation coupling caused by different levels of data augmentation.

**Table 3:** Test accuracy (%) on CIFAR-100-LT (ρ = 100) for different methods.

**Methods**	**Many**	**Medium**	**Tail**	**All**	**ECE**
BALMS (Ren et al., 2020)	–	–	–	50.8	–
PaCo (Cui et al., 2021)	62.9	53.5	35.6	51.7	9.3
BCL (Zhu et al., 2022)	**69.7**	53.8	35.5	53.9	–
RIDE (Wang et al., 2021)	66.8	53.6	23.5	49.6	34.8
SADE (Zhang et al., 2022)	66.4	51.7	29.0	50.4	17.9
Ours	69.3	**55.5**	**39.3**	**55.4**	**1.4**

All experiments used ResNet-32 as the backbone and trained for 400 epochs.

#### 5.4.2 Result for ImageNet-LT

Tables 4, 5 present the comparison results between our proposed method and existing methods on the long-tailed dataset ImageNet-LT. Compared with the multi-expert model RIDE (Wang et al., 2021) and SADE (Zhang et al., 2022), our method introduces a multiple data augmentation header with mixup based on the deep specialized feature extractor, leading to an improved performance on minority classes by effectively maintaining the model's strong representation ability from the first stage to the second stage via our proposed two-stage adjustment strategy. In contrast to other methods based on contrastive learning, such as PaCo (Cui et al., 2021) and BCL (Zhu et al., 2022), we all use various data augmentation methods. However, our proposed multi-channel deep feature extraction strategy can learn the optimal representation of different degrees of data augmentation to maximize their effectiveness. This is the main difference between our approach and others. By exploiting the different levels of data augmentation, we achieve better performance.

**Table 4:** Test accuracy (%) on ImageNet-LT on ResNet-50 and ResNetx-50 for various methods.

**Backbone**	**ResNet-50**	**ResNetx-50**
CE	47.1	48.2
MiSLAS (Zhong et al., 2021)	52.7	–
UniMix (Xu et al., 2021)	48.4	–
PaCo (Cui et al., 2021)	57.0	58.2
BCL (Zhu et al., 2022)	56.0	57.1
LA (Menon et al., 2021)	51.2	–
RIDE (Wang et al., 2021)	54.9	56.4
ACE (Cai et al., 2021)	54.8	56.5
SADE (Zhang et al., 2022)	–	58.8
Ours	**57.8**	**59.9**

**Table 5:** Test accuracy (%) on ImageNet-LT on ResNetx-50 for various methods.

**Methods**	**Many**	**Medium**	**Few**	**ALL**
LADE (Hong et al., 2021)	65.1	48.9	33.4	53.0
BL Softmax (Ren et al., 2020)	65.8	53.2	34.1	55.4
PaCo (Cui et al., 2021)	64.4	**55.7**	33.7	56.0
BCL (Zhu et al., 2022)	67.9	54.2	36.6	57.1
RIDE (Wang et al., 2021)	**68.0**	52.9	35.1	56.3
SADE (Zhang et al., 2022)	67.0	56.4	42.6	58.7
Ours	67.1	54.7	**56.5**	**59.9**

To further verify the effectiveness of our proposed reweighting method, we report the test accuracy (%) and ECE (%) on the combination of LA and different reweighting methods on ImageNet-LT using ResNet-50. All experiments used the same model structure and experimental settings as the multi-domain expert specialization model we proposed. Table 6 presents the results of our experiments, which demonstrate that our reweighting method outperformed other reweighting techniques in the minority classes, while only slightly compromising performance in the majority classes. The results suggest that appropriate reweighting methods can alleviate the overfitting of model parameters to most classes caused by the long-tailed distribution. On the other hand, inappropriate reweighting methods will lead to biased models or significant performance decreases in the majority classes.

**Table 6:** Test accuracy (%) and ECE (%) on ImageNet-LT on ResNet-50 for different reweighting methods.

**Methods**	**Many**	**Medium**	**Tail**	**ALL**	**ECE**
LA + RW	**69.3**	54.1	32.7	53.4	5.2
LA + CB	35.2	44.1	51.5	43.1	6.6
LA + FL	60.7	**53.9**	42.8	53.1	3.8
LA + Ours	65.4	52.2	**54.8**	**57.8**	**3.3**

### 5.5 Feature distribution

**Figure 5:**
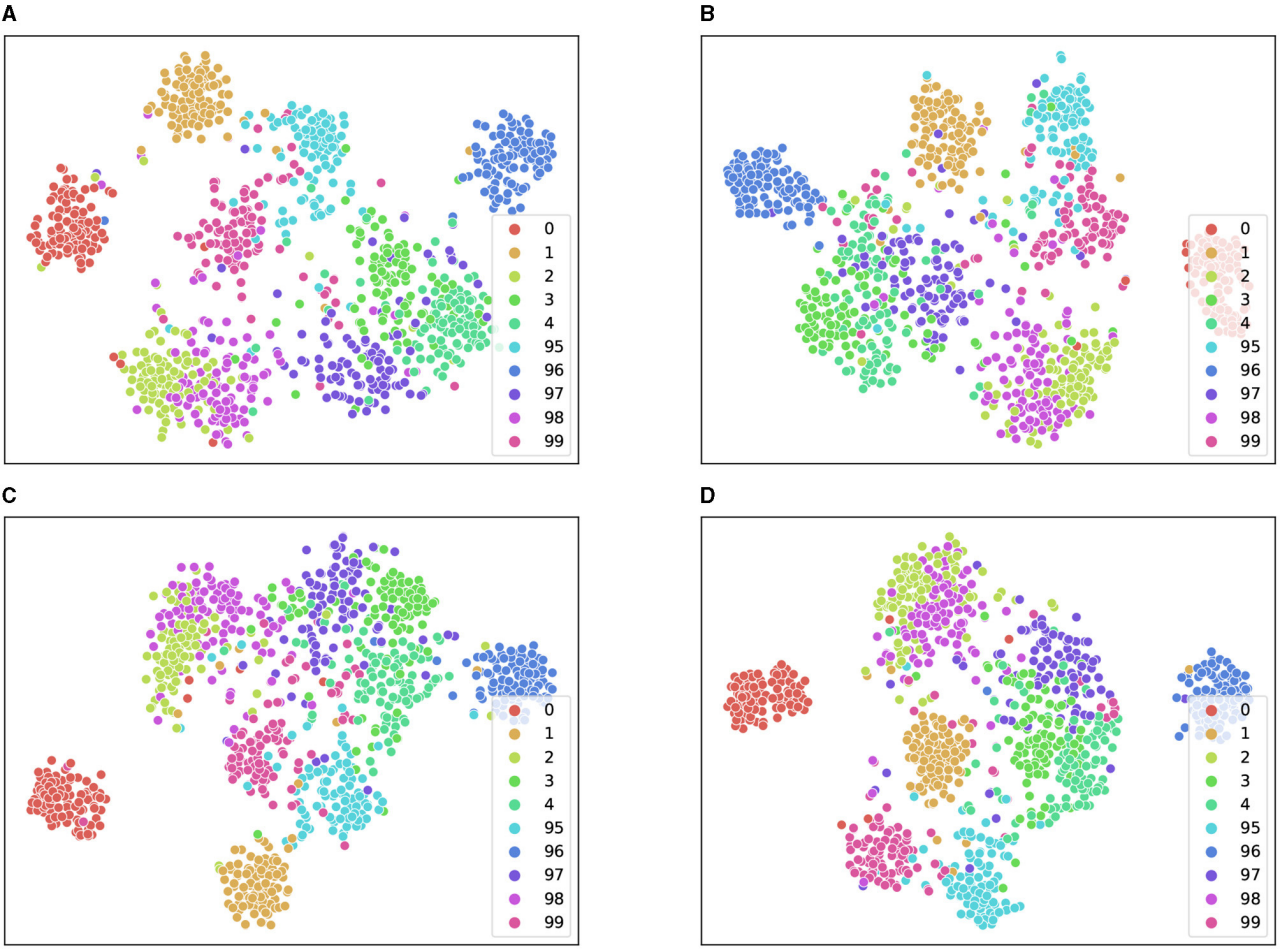
Feature distribution of the test set on CIFAR-100-LT (IR = 100). We demonstrate the distribution feature maps of t-SNE for some majority and minority classes. **(A–C)** T-SNE on three experts performing different data augmentations. **(D)** T-SNE on the mean of three experts.

To gain further insights into the effectiveness of our proposed method, we visualized the extracted features using t-SNE. As depicted in Figure 5, feature-1 and feature-2 correspond to the features obtained after dimensionality reduction. We observed that strong data augmentation could enhance feature separability but at the expense of increasing intraclass distance. By leveraging the domain expertise of three different experts and averaging their augmented features, we were able to obtain distinctive features that preserve intraclass similarity while improving interclass discrimination. This allowed us to achieve a clear decision boundary between different classes, even when using a simple linear classifier.

### 5.6 Ablation study

#### 5.6.1 The effect of different mixup parameters α

**Figure 6:**
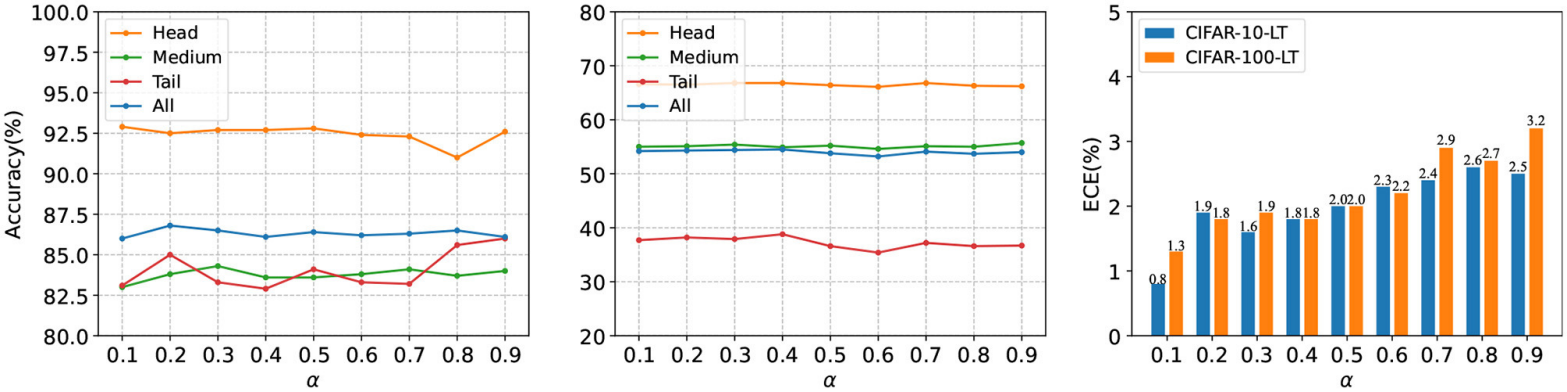
Test accuracy (%) of a ResNet-32 trained on CIFAR10-LT and CIFAR-100-LT with ρ = 100. We used a different mixup parameter, α, to conduct our experiments. **(Left)** Accuracy on CIFAR-10-LT. **(Middle)** Accuracy on CIFAR-100-LT. **(Right)** ECE (%) on CIFAR-10-LT and CIFAR-100-LT.

To study the influence of the change of mixup parameters (α) on our proposed method, we conducted a thorough ablation experiment on CIFAR-LT with ρ = 100 to find out the optimal parameter range. Figure 6 shows the result. We observed that (1) when α is > 0.4, the accuracy of the tail class fluctuates greatly; this phenomenon is obvious when the number of classes is small. The main reason for this is that with the increase in α, the value of u tends to be uniformly distributed due to the drastic change in mixing degree between different epochs and the lack of tail class data; this large randomness destroys the stability training of the tail class. (2) With an increase in α, the ECE of the results shows an increasing trend, which indicates that adjusting parameter α in our proposed method plays a crucial role in reducing the ECE of the model.

#### 5.6.2 The effect of the different hyperparameter τ

As reported in Table 7, we explored how hyperparameter τ influences the model. We can easily find that when the imbalance factor is fixed, the accuracy and ECE will decrease as the τ increases. The main reason for this phenomenon is that increasing τ enhances the effect of SLA, which changes the decision boundary while reducing intraclass spacing. As the decision boundary no longer tends to reduce overall empirical risk, this will reduce some of the model's performance.

**Table 7:** Ablation study of different imbalance ratios and τ.

		1/τ					
**Dataset**	**Imbalance ratio**	**1**	**0.9**	**0.8**	**0.7**	**0.6**	**0.5**
CIFAR-10-LT	200	83.3/1.4	83.2/1.3	83.6/1.2	83.3/1.2	83.2/0.9	83.0/0.8
	100	86.7/1.9	86.5/1.8	86.4/1.2	86.3/1.7	86.0/1.2	86.0/1.3
	50	88.9/2.4	88.3/1.9	88.6/2.5	88.4/2.0	88.8/2.2	88.5/2.0
	10	91.9/2.0	91.7/1.8	91.7/1.9	91.9/1.8	92.2/2.0	91.5/1.7
CIFAR-100-LT	200	50.1/1.8	50.1/1.6	49.9/1.6	50.2/1.4	50.1/1.2	50.0/1.0
	100	54.6/1.8	54.0/2.0	54.0/1.7	54.6/1.6	54.2/1.6	54.0/1.5
	50	58.4/1.7	58.7/2.1	58.5/1.9	58.8/1.7	58.6/1.6	58.0/1.6
	10	67.9/2.4	67.6/2.1	67.0/2.1	67.7/2.0	67.3/2.0	67.0/1.8

We chose several values from 1 to 0.5 for 1/τ to perform our ablation experiment.

#### 5.6.3 The effect of different modules

Table 8 present the results of our ablation investigation into the use of mixup in the first stage (MU), reweighting in second-stage learning (SLA), and two-stage learning (TL). As expected, we observed a decrease in accuracy and an increase in ECE for all datasets as the imbalance ratio increased. Combining MU or SLA modules with TLs consistently led to improved accuracy and reduced ECE. Notably, our proposed SLA method demonstrated a more positive impact on TL than MU under multi-data augmentations, thereby proving its effectiveness. Additionally, when all three modules were combined, our proposed algorithm maximized the model's generation ability while maintaining low ECE, despite not being optimal.

**Table 8:** Ablation study of various combinations of the module to verify the effectiveness of different modules.

**Module**			**CIFAR-10-LT**			**CIFAR-100-LT**		
**MU**	**SLA**	**TL**	**100**	**50**	**10**	**100**	**50**	**10**
		✓	84.8/5.6	87.7/3.9	91.6/3.2	53.2/6.5	58.0/6.3	67.0/4.0
✓		✓	84.7/5.4	88.2/3.4	91.5/2.8	54.1/4.0	59.2/3.0	67.3/2.8
	✓	✓	86.4/3.2	88.3/**1.3**	91.7/**1.2**	53.6/3.3	58.2/1.7	66.9/**1.2**
✓	✓	✓	**86.7**/**1.9**	**88.9**/2.4	**91.9**/2.0	**54.6**/**1.8**	**58.4**/**1.7**	**67.9**/2.4

We conducted a thorough ablation experiment. MU, using mixup in the first stage of learning; SLA, using SLA in the second stage of learning; TL, using the two-stage learning method.

## 6 Conclusion

In this study, we addressed the problem of poor model performance due to prior shift and covariate shift caused by imbalanced distribution. To investigate the impact of logit adjustment and reweighting on model performance, we employed the two-stage learning method, which is currently a popular research direction. Our analysis revealed that combining existing reweighting methods and logit adjustment not only reduces model performance but also increases ECE. Therefore, we proposed a sample logit-aware reweighting method that assigns more suitable weights to hard samples from majority classes and samples from minority classes. Additionally, to tackle the covariate shift problem, we introduced a multi-domain expert specialization model designed to enhance the feature extraction ability of the model. Through experiments conducted on various datasets, we demonstrated the effectiveness of our proposed method. Furthermore, ablation experiments reinforced our findings and emphasized that our proposed model outperforms current state-of-the-art methods. Overall, our study highlights the necessity of addressing prior and covariate shift in imbalanced datasets and provides an effective solution to improve model performance.

## Data availability statement

Publicly available datasets were analyzed in this study. This data can be found here: https://www.cs.toronto.edu/~kriz/cifar-10-python.tar.gz; https://www.cs.toronto.edu/~kriz/cifar-100-python.tar.gz; https://image-net.org/.

## Author contributions

NL: Formal analysis, Software, Writing—original draft. JW: Writing—review & editing. YZ: Data curation, Writing—review & editing. LW: Writing—review & editing. QL: Funding acquisition, Supervision, Writing—review & editing.

## Appendix

### Three different image augmentations

CIFAR-LT: For small augmentation, we used random crop (which randomly crops 32 × 32 pixels in the image) with a padding of four pixels and a random horizontal flip. For medium augmentation, we used RandomResizedCrop (the crop size of which is the same as random crop) with scale (0, 2, 1), random horizontal flip, and the same setting with SimCLR (Chen et al., 2020), which includes random gray, random GaussianBlur, and random ColorJitter. For strong augmentation, we used random crop (which randomly crops 32 × 32 pixels in the image) with a padding of four pixels, random horizontal flip, and CIFAR-Policy (Cubuk et al., 2018).

ImageNet-LT: For small augmentation, we used RandomResizedCrop (which randomly crop the image and resized it to 224 × 224 pixels in the image), random horizontal flip, and random ColorJitter. For medium augmentation, we used RandomResizedCrop, random horizontal flip, and the same setting with SimCLR (Chen et al., 2020). For strong augmentation, we used RandomResizedCrop and random horizontal flip with ImageNet-Policy (Cubuk et al., 2018).
